# Management of asymmetrical presentation of non-syndromic mandibular supernumerary teeth: a case report

**DOI:** 10.1097/MS9.0000000000001926

**Published:** 2025-05-30

**Authors:** Adetola Babalola, David Adetula, Kudirat Giwa, Adeyemi Falegan

**Affiliations:** a Ever-Smiling Dental Clinic, Ibadan, Nigeria; b Faculty of Dentistry, University of Ibadan and University College Hospital, Ibadan, Nigeria; c Royal College of Dental Surgeon, Ontario, California

**Keywords:** asyndromic, exodontia, hyperdontia, supernumerary teeth

## Abstract

**Introduction and importance of hyperdontia:**

Hyperdontia, characterized by the presence of supernumerary teeth, is a developmental anomaly with potential clinical implications. Understanding its prevalence, classifications, and associated complications is crucial for early diagnosis and effective management.

**Case presentation:**

This report outlines the case of a 25-year-old lady with a history of extra teeth intruding into her tongue space. The examination revealed bilateral asymmetrical supernumerary teeth in the mandibular premolar region, a presentation not commonly reported. The patient sought expert advice and management due to the familial occurrence of a similar condition.

**Clinical discussion:**

The presented case deviates from typical reports, being non-syndromic, bilateral, and asymmetrical in supernumerary tooth number. The patient exhibited good oral hygiene and a complete permanent set of teeth in the maxillary arch. In the mandibular arch, fully erupted para premolars were identified, as non-carious, non-mobile, and not fused to any other tooth. Simple exodontia with minimal invasiveness was employed as the treatment modality. The procedure, performed under local anesthesia, involved surgical extraction using forceps and Coupland elevators. Postoperative care included hemostasis, digital reduction of extraction sockets, and prescription of medications. Follow-up assessments and a postoperative orthopantomogram were scheduled to rule out unerupted supernumerary teeth or odontoma.

**Conclusion:**

Following the Surgical CAse REport Guidelines criteria, this case report contributes to understanding hyperdontia variations, emphasizing the importance of early diagnosis and intervention. The non-syndromic, bilateral, and asymmetrical nature of the case challenges existing reports, highlighting the need for diverse data in dental literature, especially from regions like Africa.

## Introduction

Hyperdontia, or supernumerary teeth (ST), is the presence of excess teeth, either erupted or unerupted, outside of the regular 32 permanent teeth in an adult or 20 primary teeth in a pediatric^[1]^. ST often appear unilaterally. Rarely can they appear bilaterally and asymmetrically. Mesiodens is the most common type of ST. Then, the upper fourth molars and lower small molars follow in order. In deciduous dentition, ST most commonly occur in incisors. They rarely appear in deciduous canines^[2]^. ST have multiple classifications. They can be classified based on chronology, location, morphology, and orientation^[3]^. The extra teeth can be present anywhere in the maxilla or mandible and can be single or multiple. In the primary dentition, morphology is usually normal or conical. There is a greater variety of forms presenting in the permanent dentition. Four different morphological types of ST have been described: conical, tuberculate, supplemental, and odontoma^[4]^.

**HIGHLIGHTS**

- Two on the left and one on the right hence asymmetrical in number.
- There was no history of any medical condition which ruled out any systemic medical conditions or syndromes.
- Simple and minimally invasive exodontia was used to manage the case.
- There was no complication and all sockets were fully healed by 1 week of follow-up.

Most studies report that the prevalence of ST ranges between 0.1% and 3.8% in permanent dentition and 0.35%–0.6% in primary dentition^[5,6]^. Recent studies by Irish^[6]^ reported a 3.08% hyperdontia prevalence among all 1429 modern sub-Saharan Africans. ST are rarely present in the same individual’s deciduous and permanent dentitions. They can remain unerupted with permanent teeth, cause dental crowding, and sometimes prevent the eruption of permanent teeth. If they remain impacted, they can cause teeth displacement and root resorption. Supernumerary deciduous teeth can cause delayed development or vestibuloversion of normal deciduous teeth^[7]^. Complications associated with ST include impaction, delayed eruption, ectopic eruption, dental overcrowding, teeth spatial disorders, and the formation of follicular cysts^[8]^. ST are usually associated with disorders such as cleft lip and cleft palate or syndromes such as Gardner syndrome, Down syndrome, cleidocranial dysplasia, Zimerman-Laby syndrome, or Noonan syndrome^[9]^.

The etiology of ST has yet to be understood entirely. Both genetic and environmental factors have been considered^[10]^. Several theories have been suggested to explain their occurrence^[11]^. Some of these theories include Atavism; it was initially told that ST resulted from phylogenetic reversion to extinct primates with three pairs of incisors. This theory has been primarily discounted^[12]^. Dichotomy theory states that the tooth bud splits into two equal or different-sized parts, resulting in two teeth of similar size or one regular and one dysmorphic tooth, respectively^[13]^. However, this theory has also been discounted^[12]^.

We also have the Dental lamina hyperactivity theory; according to this theory, a supplemental form would develop from the lingual extension of an accessory tooth bud, whereas a rudimentary form would develop from the proliferation of epithelial remnants of the dental lamina. Although all theories are hypothetical because of the inability to obtain sufficient embryological material, most literature supports the dental lamina hyperactivity theory^[12]^.

Finally, there are genetic factors: These are considered necessary in the occurrence of ST. A sex-linked inheritance has been suggested by the observation that males are affected approximately twice as often as females^[12]^.

## Case report

This is a case of a 25-year-old lady who presented at the dental facility in the sub-urban area of Ibadan, Nigeria. Her primary complaint was having extra teeth intruding into her tongue space. The history of complaints revealed that the teeth had been present since she developed her permanent teeth in secondary school (about 13 years before the presentation). Her medical/drug histories were not contributory. She gave a positive history of a similar condition in her father, who had told her it was common in the family. She had no other complaints and wanted expert advice and management.

On general examination, she was healthy-looking with no apparent clinical abnormality on a thorough inspection. Intraoral examination revealed good mouth opening and oral hygiene, evidenced by no debris, plaque, or calculus. The tongue, buccal mucosal, and floor of the mouth also appeared clinically normal. Hard tissue examination showed a complete permanent set of teeth in the maxillary arch. On the mandibular arch, there were fully erupted para premolars.

**Figure 1.**
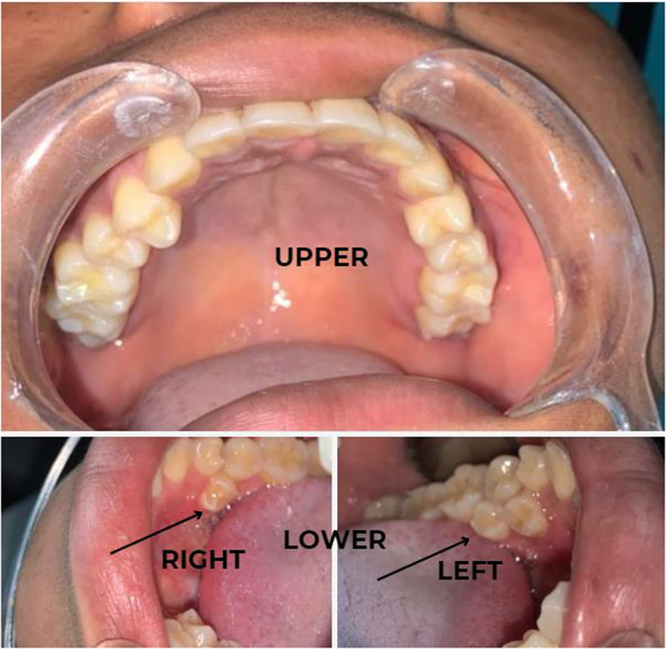
Intraoral photograph showing the supernumerary teeth.

Two are on the right, and one is on the left (Fig. 1). They were non-carious and non-mobile. They were also not fused to any other tooth.

## Management approach

Different treatment modalities have been discussed, including surgical extractions, esthetic management, and orthodontic treatment of the ST. It is essential to note that treatment of ST depends on their type, position, and complications, identified clinically and radiographically. Following the Surgical CAse REport Guidelines criteria^[14]^, a detailed case report is reported.

In our study, the minimally invasive simple exodontia technique was utilized by a skilled general dentist. The procedure was explained to the patient and written, and verbal/written informed consent was obtained in the presence of three dental nurses who also served as chaperones. Local anesthesia (LA) (2% lidocaine with 1:100 000 adrenaline) was delivered. Subjective and objective tests were done to assess the onset of action of the LA. After proper anesthesia, exodontia was performed using forceps and Coupland elevators. Since a simple rotatory and minimally invasive extraction technique was utilized, hemostasis was easily achieved using gauze and cotton rolls. Digital reduction of the extraction sockets was also done. Postoperative instructions (verbal and written) were given. Finally, postoperative medications were prescribed.

**Figure 2.**
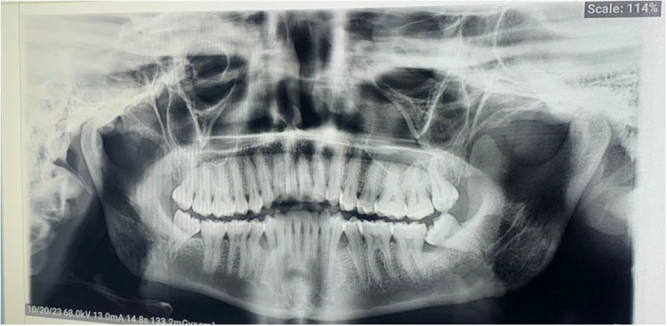
Postoperative orthopantomogram.

**Figure 3.**
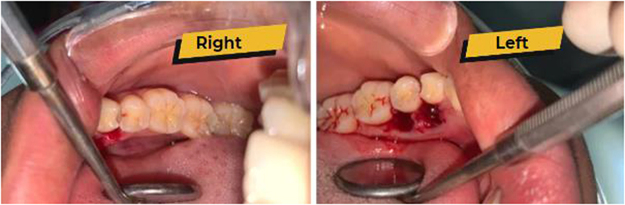
Immediate postoperative photograph.

**Figure 4.**
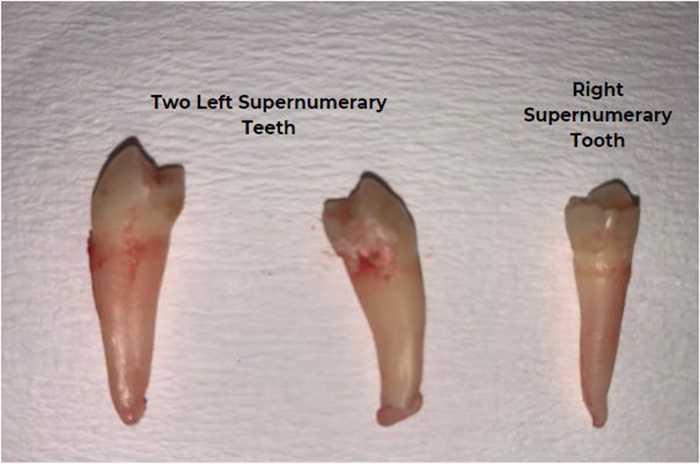
Full images of the extracted supernumerary teeth.

**Figure 5.**
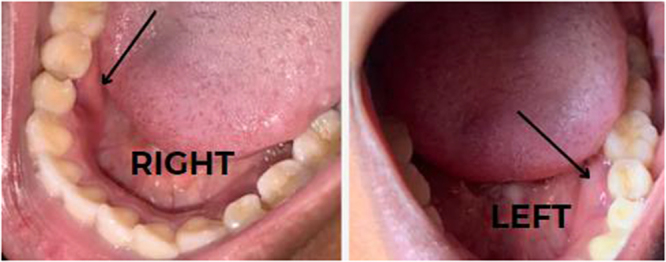
One week post-extraction socket.

Due to lack of access to an orthopantomogram (OPG) as at presentation, the radiograph could not be taken; however, a postoperative OPG was requested to rule out unerupted ST or odontoma (Fig. 2). Periapical radiographs would have shown insignificant findings due to the lingual locations of the ST. We took immediate postoperative pictures (Fig. 3). Also, the images of the extracted ST revealed their whole roots and conical shapes (Fig. 4). One week after the procedure, there was a follow-up, and the patient had no complications. Finally, an image of the healing socket after 1 week is shown in Figure 5.

## Discussion

Hyperdontia is a developmental dental anomaly of number, referring to any excess dental or odontological structure^[6]^, formed from tooth germ more than the usual number for any given region of the dental arch”^[15]^ that is not a part of the normal dentition^[16]^. Hyperdontia usually involves few teeth: about 76%–86% of patients have only one supernumerary tooth, while about 12%–23% will have only two ST^[8]^. It has been reported that 60.9% occurred in the mandible, and among these, 44.8% occurred in the mandibular premolar region^[17,18]^, similar to our case report.

Only 1% of non-syndromic cases present multiple ST, which occur more frequently in the mandibular premolars and in the anterior region^[19]^; numerous STs have been found to occur more regularly in the premolar region^[20]^, which also follows the same pattern with our patient. In a study carried out in a Nigerian center, 27 subjects (84.3%) had unilateral hyperdontia. In contrast, multiple and bilateral hypodontia were observed in 9.4% (*n* = 3) and 6.3% (*n* = 2) of total subjects, respectively, which is in contrast with reports of supernumerary being bilateral in their findings^[5]^ – our finding, although a bilateral presentation, is asymmetrical in number. Khalaf *et al*^[18]^ have reported various presentations in the premolar region. However, we have further shown how a minimally invasive and simple exodontia technique was used to manage our case, especially in a low-resource setting.

Treatment of ST includes several controversies and varied opinions among authors, particularly concerning the timing of removal^[17]^. The usual treatment is to extract the supernumerary tooth, although repositioning it in the dental arch may be an alternative option^[8]^. Treatment may vary from just extraction of ST or extraction followed by orthodontic correction to establish a good occlusion. There is no consensus on the best time for surgical extraction of an unerupted supernumerary tooth^[17]^.

Högström and Andersson^[21]^ suggested two options. The first option involves the removal of the supernumerary as soon as it has been diagnosed. This could create dental phobia in a young child and cause devitalization or deformation of adjacent teeth. Second, the supernumerary could be left until the root development of the adjacent teeth is complete^[17]^. Since an unerupted supernumerary tooth can be associated with many complications, such as disruption of occlusion and periodontal diseases due to overcrowding, it should be surgically removed as was done in our case.

## Conclusion

The awareness of hyperdontia pattern/prevalence can be helpful in early diagnosis and prevention by general practitioners, pediatric dentists, and orthodontists. Since most (80%–93%) of ST can cause clinical complications, their early diagnosis and orthodontic/surgical intervention significantly reduce the clinical problems of adjacent permanent teeth and install the occlusion^[18]^. In addition, our case is peculiar because it does not correlate with what is being reported. It is a non-syndromic, bilateral but asymmetrical in number, with multiple para premolar ST. Although few case reports from Africa have reported maxillary and pre-maxilla cases. This will contribute to African data regarding variation in presentation and management using simple and minimally invasive exodontia.

## Data Availability

Data sharing is not applicable to this article.

## References

[1] Anthonappa RP King NM Rabie ABM. Aetiology of supernumerary teeth: a literature review. Eur Arch Paediatr Dent 2013;14:279–88. 24068489 10.1007/s40368-013-0082-z

[2] Ratson T Peretz B. Non-syndromic multiple hyperdontia in monozygotic twin sisters: a report of two cases. J Dent Child 2014;81:50–53. 24709435

[3] Cheng F-C Chen M-H Liu B-L. Nonsyndromic supernumerary teeth in patients in National Taiwan University Children’s Hospital. J Dent Sci 2022;17:1612–18. 36299357 10.1016/j.jds.2022.07.015 PMC9588821

[4] Garvey MT Barry HJ Blake M. Supernumerary teeth–an overview of classification, diagnosis and management. J Can Dent Assoc 1999;65:612–16. 10658390

[5] Bello S Olatunbosun W Adeoye J. Prevalence and presentation of hyperdontia in a non-syndromic, mixed Nigerian population. J Clin Exp Dent 2019;11:e930–6. 31636863 10.4317/jced.55767 PMC6797466

[6] Irish JD. Hyperdontia across sub-Saharan Africa: prevalence, patterning, and implications. Arch Oral Biol 2022;140:105463. 35617756 10.1016/j.archoralbio.2022.105463

[7] Eshgian N Al-Talib T Nelson S. Prevalence of hyperdontia, hypodontia, and concomitant hypo-hyperdontia. J Dent Sci 2021;16:713–17. 33854723 10.1016/j.jds.2020.09.005 PMC8025189

[8] Ata-Ali F Ata-Ali J Peñarrocha-Oltra D. Prevalence, etiology, diagnosis, treatment and complications of supernumerary teeth. J Clin Exp Dent 2014;6:e414–8. 25593666 10.4317/jced.51499 PMC4282911

[9] Bayar GR Ortakoglu K Sencimen M. Multiple impacted teeth: report of 3 cases. Eur J Dent 2008;2:73–78. 19212513 PMC2633158

[10] Hall A Onn A. The development of supernumerary teeth in the mandible in cases with a history of supernumeraries in the pre-maxillary region. J Orthod 2006;33:250–55. 17142331 10.1179/146531205225021735

[11] Primosch RE. Anterior supernumerary teeth – assessment and surgical intervention in children. Am Acad Pedod 1980;3:204–15. 6945564

[12] Srinivasan K. Mystery behind hyperdontia: report of two cases. Int J Appl Dent Sci 2015;1:05–9.

[13] Benazzi S Fantini M De Crescenzio F. Improving the spatial orientation of human teeth using a virtual 3D approach. J Hum Evol 2009;56:286–93. 19167741 10.1016/j.jhevol.2008.07.006

[14] Sohrabi C Mathew G Maria N. The SCARE 2023 guideline: updating consensus Surgical CAse REport (SCARE) guidelines. Int J Surg 2023;109:1136–40. 37013953 10.1097/JS9.0000000000000373 PMC10389401

[15] Arathi R Ashwini R. Supernumerary teeth: a case report. J Indian Soc Pedod Prev Dent 2005;23:103–05. 16012216 10.4103/0970-4388.16453

[16] Moradinejad M Hashemi Ashtiani A Rakhshan V. Multiple nonsyndromic unerupted supernumerary teeth: a report of a rare case. Case Rep Dent 2022;2022:4063856. 35392488 10.1155/2022/4063856 PMC8983268

[17] Amarlal D Muthu MS. Supernumerary teeth: review of literature and decision support system. Indian J Dent Res 2013;24:117–22. 23852244 10.4103/0970-9290.114911

[18] Khalaf K Al Shehadat S Murray CA. A review of supernumerary teeth in the premolar region. Int J Dent 2018;2018:6289047. 30631362 10.1155/2018/6289047 PMC6304893

[19] Amini F Rakhshan V Jamalzadeh S. Prevalence and pattern of accessory teeth (hyperdontia) in permanent dentition of Iranian orthodontic patients. Iran J Public Health 2013;42:1259–65. 26171338 PMC4499067

[20] Belmehdi A Bahbah S El Harti K. Non syndromic supernumerary teeth: management of two clinical cases. Pan Afr Med J 2018;29:163. 30050627 10.11604/pamj.2018.29.163.14427 PMC6057571

[21] Högström A Andersson L. Complications related to surgical removal of anterior supernumerary teeth in children. ASDC J Dent Child 1987;54:341–43. 3478360

